# First report of haemosporidia and associated risk factors in red junglefowl (*Gallus gallus*) in China

**DOI:** 10.1186/s13071-022-05389-2

**Published:** 2022-08-01

**Authors:** Zhao Li, Xiao-Xia Ren, Yin-Jiao Zhao, Lian-Tao Yang, Bo-fang Duan, Na-Ying Hu, Feng-Cai Zou, Xing-Quan Zhu, Jun-Jun He, Qi-Shuai Liu

**Affiliations:** 1 Animal Research and Resource Center, Yunnan University, Kunming, Yunnan Province 650500 People’s Republic of China; 2 State Key Laboratory of Conservation and Utilization of Bio-Resources in Yunnan, Center for Life Science, School of Life Sciences, Yunnan University, Kunming, Yunnan Province 650500 People’s Republic of China; 3 Yunnan Province Center for Animal Disease Control and Prevention, Kunming, Yunnan Province 650201 People’s Republic of China; 4 Xishuangbanna Dai Autonomous Prefecture Technical Extension Station for Animal Husbandry and Veterinary Medicine, Jinghong, Yunnan Province 666100 People’s Republic of China; 5 Key Laboratory of Veterinary Public Health of Yunnan Province, College of Veterinary Medicine, Yunnan Agricultural University, Kunming, Yunnan Province 650201 People’s Republic of China; 6 College of Veterinary Medicine, Shanxi Agricultural University, Taigu, Shanxi Province 030801 People’s Republic of China

**Keywords:** Avian haemosporidia, Prevalence, *cytb*, Red junglefowl, China

## Abstract

**Background:**

Avian haemosporidia infect both domestic and wild birds, causing anemia, acute tissue degeneration, and depopulation in wild birds. Poultry and wild birds have been reported as common reservoirs of haemosporidia, but limited information is available for red junglefowl (*Gallus gallus*) in China. The present study investigated the prevalence and molecular characterization of haemosporidia in red junglefowl.

**Methods:**

Blood samples were collected from 234 red junglefowl from Jinghong City of Yunnan Province, and genomic DNA was extracted from these samples. The prevalence of haemosporidia was determined by nested PCR targeting the mitochondrial cytochrome *b* (*cytb*) gene. Molecular characterization was investigated based on phylogenetic analysis of *cytb* sequences, and associated risk factors were analyzed using the Chi-square (*χ*^2^) test.

**Results:**

The overall prevalence of haemosporidia was 74.8% (175/234), and three species were identified, namely *Haemoproteus enucleator*, *Leucocytozoon californicus*, and *Plasmodium juxtanucleare*. The prevalence of haemosporidia in adult fowl (81.1%, 107/132) was significantly higher (*χ*^2^ = 6.32, *df* = 1, *P* = 0.012) than that in juveniles (66.7%, 68/102). Three novel haemosporidian lineages were revealed.

**Conclusions:**

This study examined the prevalence and identified species of avian haemosporidians in red junglefowl, providing new information on the molecular epidemiology and geographical distribution of haemosporidian parasites. Our results indicated high prevalence and diverse species distribution of these haemosporidians in red junglefowl. To the best of our knowledge, this is the first record of haemosporidian infection in red junglefowl in China.

**Graphical Abstract:**

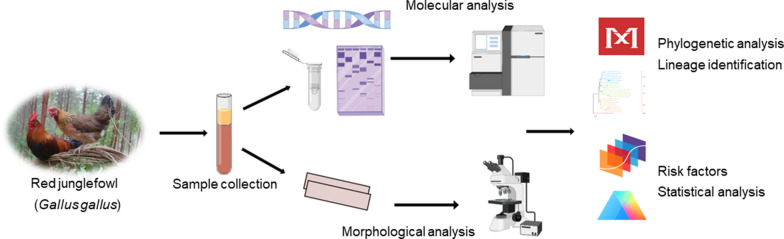

## Background

Avian haemosporidia are a whole group of organisms containing hundreds of species [1]. The group has been used for decades as a model to study the mechanisms of disease transmission and interspecific co-evolution [2]. Haemosporidia of the genera *Plasmodium*, *Haemoproteus*, and *Leucocytozoon* are diverse groups of vector-transmitted blood parasites that are abundant in most avian families and can cause disease [2–4]. At present, there are more than 4000 lineages defined based on the barcode sequence of the mitochondrial cytochrome *b* gene (*cytb*). Approximately 2000 bird species can be infected by haemosporidia, and these parasites have been found in all regions of the world except Antarctica, posing a serious threat to the health and even survival of infected poultry and birds [5].

Over the many centuries since the domestication of chickens, they have been respected by different cultures all over the world. Compared with sheep, cattle, pigs, and other livestock, chicken is the preferred source of animal protein. Red junglefowl (*Gallus gallus*) has been identified as the wild ancestor of domestic chicken (*Gallus gallus domesticus*) [6]. Due to the warm and humid tropical rain forest climate, Xishuangbanna is rich in biodiversity, which is highly suitable for domestic chickens and their insect vectors. Avian haemosporidia are mainly transmitted by dipteran-blood sucking insects such as mosquitoes, biting midges, and black flies [7, 8]. In poultry, haemosporidiosis can lead to clinical manifestations such as multiple organ injury, anemia, and weight loss, which seriously affects the economic benefits of poultry breeding [9, 10]. Failure to provide timely preventive treatment will lead to higher rates of infection and mortality [11, 12].

Information about patterns of distribution of haemosporidia in poultry contributes to better prevention, control, and treatment of avian haemosporidiosis. However, to date, there are limited studies on haemosporidian infection in red junglefowl. Therefore, the main objectives of the present study were to investigate the prevalence, molecular characterization, and associated risk factors of haemosporidia in red junglefowl using molecular biology and high-throughput sequencing, evaluating the factors associated with haemosporidian infection in red junglefowl using cross-sectional analysis.

## Methods

### Sample collection

The red junglefowl is a tropical member of the pheasant family and the direct ancestor of the domestic chicken. With the help of the staff of Yunnan Province Center for Animal Disease Control and Prevention, Xishuangbanna Dai Autonomous Prefecture Technical Extension Station for Animal Husbandry and Veterinary Medicine, from November 2020 to May 2021, a total of 234 blood specimens were collected from red junglefowl in a tea plantation habitat in Jinghong City (21°27′ ~ 22°36′N, 100°25′ ~ 101°31′E), Yunnan Province, southwestern China. These domestic chickens were divided into two age groups: juveniles and adults. Samples were divided into three groups according to body weight: < 0.5 kg, 0.5–1.0 kg, and > 1 kg. Each fresh blood specimen was randomly obtained from the inferior pterygoid vein of each apparently healthy fowl using a vacuum blood collection tube with anticlotting agents including ethylenediaminetetraacetic acid (EDTA). The vacuum blood collection tubes containing approximately 2–4 ml individual animal blood samples were then labeled with sex, weight, age, sampling site, and sampling time, and immediately kept on ice packs at −80 °C during transport.

### Molecular analysis

The genomic DNA of each blood sample was extracted using a commercial DNA kit (Tiangen Bio-tech Co., Ltd, Beijing, China) according to the manufacturer’s instructions. The extracted genomic DNA was stored at −20 °C for further polymerase chain reaction (PCR) analysis. Avian haemosporidian infection in red junglefowl was detected by nested PCR amplification of a 479-base-pair fragment of the mitochondrial *cytb* gene using primers and procedures described previously [13]. For the first PCR, the primers HaemNFI (5′-CATATATTAAGAGAAITATGGAG-3′) and HaemNR3 (5′-ATAGAAAGATAAGAAATACCATTC-3′) were used. In the second PCR, two primer pairs were applied: the primers HaemNF (5′-ATGGTGCTTTCGATATATGCATG-3′) and HaemNR2 (5′-GCATTATCTGGATGTGATAATGGT-3′), and HaemNFL (5′-ATGGTGTTTTAGATACTTACATT-3′) and HaemNR2L (5′-CATTATCTGGATGAGATAATGGIGC-3′). Amplification products were tested by running 2 μl of the second PCR product on 1.5% agarose gel stained with SYBR Green I and visualized with UVP GelStudio DNA Gel Documentation Imaging Systems (Analytik Jena Company, US, https://www.laboratory-equipment.com/uvp-gelstudio-dna-gel-documentation-systems-analytik-jena.html). One negative control (nuclease-free water) and three positive controls were used to determine possible false amplifications.

### Bioinformatics, lineage identification, and phylogenetic analysis

All positive secondary PCR products were purified and sequenced by Kunming Sangon Biotech (Shanghai) Co., Ltd. Sequences obtained were firstly proofread with their DNA peak-form graph using Chromas 2.6. Using MEGA X (Version 10.2.6, https://www.megasoftware.net/), the sequences of amplification products were aligned with the most similar lineages according to the BLAST result in the MalAvi database (http://130.235.244.92/Malavi/blast.html) [5, 14]. Haplotypes were defined as new lineages if they differed by one base pair from lineages deposited in the MalAvi database (http://mbio-serv2.mbioe kol.lu.se/Malavi). The phylogenetic analysis was performed using the neighbor-joining (NJ) method with MEGA X; the Kimura 2-parameter model was selected, and 1000 bootstrap replicates were applied in this study. The numbers at the nodes indicate the bootstrap support obtained by repeating the analysis 1000 times, and values above 50% are shown.

### Statistical analysis

The prevalence of avian haemosporidian parasites among different red junglefowl groups according to sex, age, weight, and sampling season were calculated by Chi-square (*χ*^2^) tests using SPSS 22.0 (IBM Corporation, https://www.ibm.com/cn-zh), and were considered statistically significant if *P* < 0.05. The odds ratios (ORs) and their 95% confidence intervals (95% CIs) were calculated and analyzed by using GraphPad Prism Version 8.02 for Windows (GraphPad Software, Inc., https://www.graphpad.com/).

## Results

### Prevalence of avian haemosporidia in red junglefowl

**Fig. 1:**
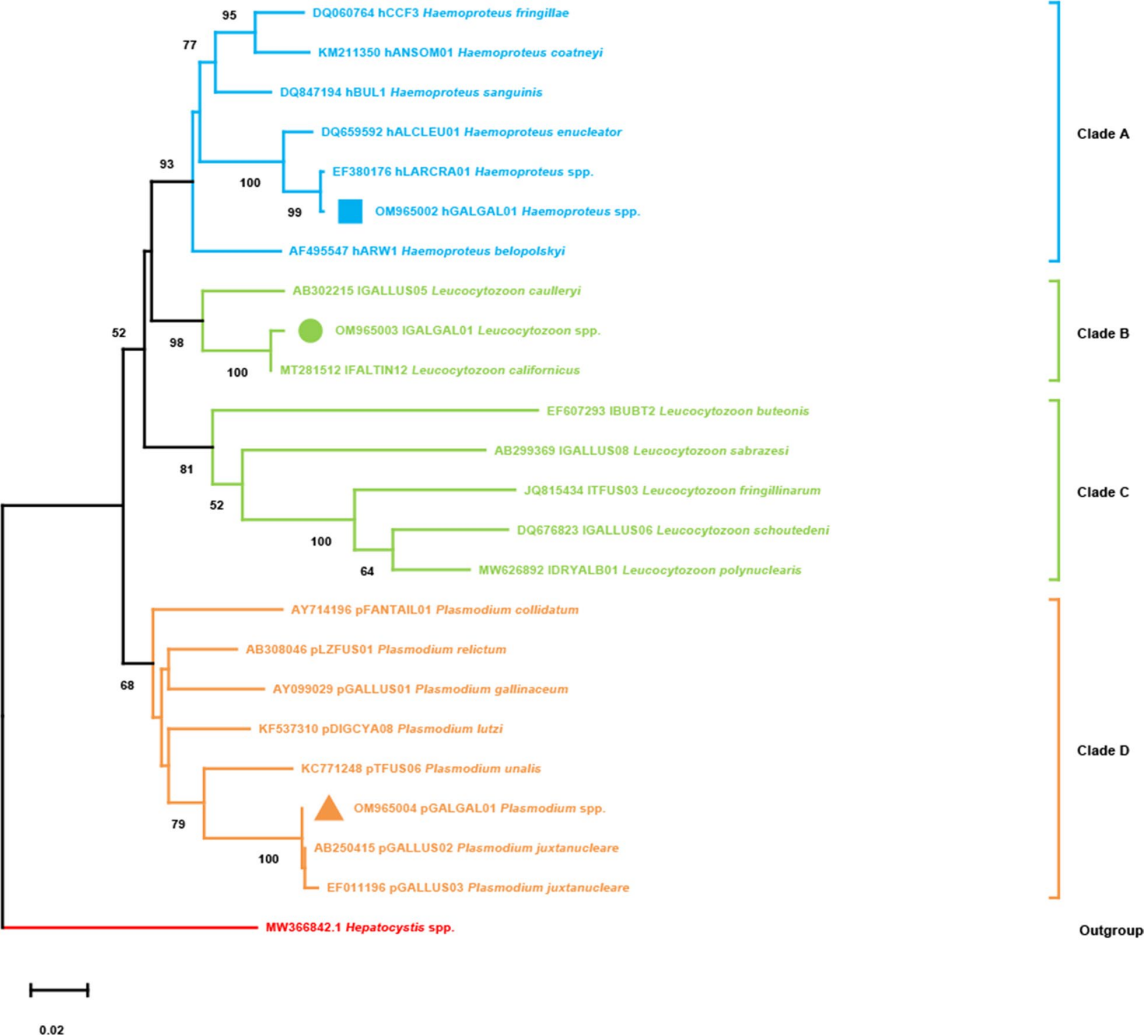
Phylogenetic tree of avian haemosporidia (*Plasmodium*, *Haemoproteus*, and *Leucocytozoon*) based on *cytb* sequences. One lineage of *Hepatocystis* spp. was used as an outgroup. Parasite species names and GenBank accession numbers are provided in the tree. The parasite lineages reported in this study are marked by blue squares, green dots, and yellow triangles, respectively. The bootstrap value is shown when the value is greater than 50%

Haemosporidia belonging to the genera *Haemoproteus*, *Plasmodium*, and *Leucocytozoon* were detected in red junglefowl (Tables 1 and 2, Fig. 1). As shown in Table 1, 175 out of 234 DNA samples were positive for avian haemosporidia, representing a 74.8% overall prevalence.

**Table 1:** Prevalence of avian haemosporidia (*Plasmodium*, *Haemoproteus*, and *Leucocytozoon*) in blood samples from red junglefowl (*Gallus gallus*) in Yunnan Province, southwestern China

Variable	No. positive/tested	Prevalence (95% CI)	Odds ratio (95% CI)	*P*-value
Sex				
Female	123/167	73.7 (67.0–80.3)	Reference	0.528
Male	52/67	77.6 (67.6–87.6)	0.81 (0.41–1.58)	
Age				
Juvenile	68/102	66.7 (57.5–75.8)	Reference	0.012
Adult	107/132	81.1 (74.4–87.7)	0.47 (0.26–0.58)	
Weight				
< 0.5 kg	32/48	66.7 (53.3–80.0)	Reference	0.149
0.5–1.0 kg	87/118	73.7 (65.8–81.7)	0.71 (0.35–1.47)	
> 1 kg	56/68	82.4 (73.3–91.4)	0.43 (0.18–1.02)	
Seasons				
Summer	106/131	80.9 (74.2–87.6)	Reference	0.015
Winter	69/103	67.0 (57.9–76.1)	2.09 (1.15–3.80)	
Total	175/234	74.8 (69.2–80.5)

**Table 2:** Infection information, parasite species, and lineage of avian haemosporidia (*Plasmodium*, *Haemoproteus*, and *Leucocytozoon*) in red junglefowl (*Gallus gallus*) in Yunnan Province, China

Infection type	Parasite Genus	No. positive	Proportion %	Parasite species	Lineage name
Single infected	H	7	4.0	*H. enucleator*	hGALGAL01
	L	32	18.3	*L. californicus*	lGALGAL01
	P	114	65.1	*P. juxtanucleare*	pGALGAL01
Subtotal		153	87.4		
Mixed infected	H, L	3	1.7	*H. enucleator*	hGALGAL01
				*L. californicus*	lGALGAL01
	H, P	3	1.7	*H. enucleator*	hGALGAL01
				*P. juxtanucleare*	pGALGAL01
	L, P	10	5.7	*L. californicus*	lGALGAL01
				*P. juxtanucleare*	pGALGAL01
	H, L, P	6	3.4	*H. enucleator*	hGALGAL01
				*L. californicus*	lGALGAL01
				*P. juxtanucleare*	pGALGAL01
Subtotal		22	12.6		
Total		175/234

Among samples positive for haemosporidian infection, 107 were in adult fowls, with an infection rate of 81.1% (107/132), while the infection rate in juveniles was 66.7% (68/102). A significant difference was observed between the two age groups (*χ*^2^ = 6.32, *df* = 1, *P* = 0.012). The positive rate of blood samples collected in summer (80.9%, 106/131) was higher than that in winter (67.0%, 69/103). According to Chi-square tests, we identified the risk factors for the prevalence of haemosporidia in fowls as age (OR 0.47, 95% CI 0.26–0.58, *P* 0.012) and season (OD 2.09, 95% CI 1.15–3.80, *P* 0.015).

We found single and mixed haemosporidian infections in red junglefowl (Table 2). Of 175 blood samples that tested positive by the PCR technique, 153 (153/175, 87.4%) samples were single pathogen infections, of which seven samples were *Haemoproteus* infections, 32 were *Leucocytozoon* infections, and 114 were *Plasmodium* infections. In addition, there were 22 (22/175, 12.6%) samples which were mixed infections, with 16 samples infected with two pathogens and six samples infected with three pathogens.

### Molecular characterization of avian haemosporidia

Molecular analysis revealed parasites belonging to three different genera: *Haemoproteus*, *Plasmodium*, and *Leucocytozoon* (Fig. 1). The three lineages of haemosporidia clustered with their genetically most similar lineages within the corresponding parasite genera. Our three representative lineages hGALGAL01, lGALGAL01, and pGALGAL01 were more similar to *H. enucleator*, *L. californicus*, and *P. juxtanucleare*, respectively (Fig. 1).

## Discussion

The global prevalence of haemosporidia in red junglefowl was 74.8% (175/234), which is much higher than that of fighting cocks from Thailand (20.8%, 52/250) [15], but lower than that in domestic chickens from Nan, Prachinburi, and Chachoengsao provinces of Thailand (79.6%, 125/157) [16] and in indigenous chickens from the north central part of Nigeria (75.0%, 81/108) [17]. The reason for this may be the abundance of vegetation in tropical areas, with species of *Culicoides* and avian haemosporidia transmitted by biting midges and other insect vectors [18, 19]. In addition, the reason for the variation in prevalence is complicated, and many factors will affect the detection rate, such as sampling time, age group, sampling number, and geographical conditions [20]. In addition, similar to previous studies, the proportion of single infection was much higher than that of mixed infections [21, 22], and mixed infections showed multiple combinations [23, 24].

Avian haemosporidia were detected in juvenile and adult fowls with infection rates of 66.7% (68/102) and 81.1% (107/132) (*P* 0.012), respectively. Previous studies showed that infection rates were higher in young birds relative to adults, possibly due to the lower immune resistance in young birds [25, 26]. The greater area of bare skin of young domestic chickens makes them more easily accessible to the pathogen vectors [27]. The weight of red junglefowl did not appear to contribute significantly to *Haemoproteus* spp. infection. It is true that many studies have shown that different host traits and abiotic factors are important determinants in a host–parasite interaction [28, 29]. Factors such as plant richness, vector species, temperature, and humidity in wild bird habitats contribute significantly to the prevalence and diversity of *Haemoproteus* spp. [30- 32].

Avian haemosporidia in birds is genetically diverse [33, 34]. The representative *Haemoproteus* gene (accession no. OM965002) is closely related to *Haemoproteus* spp. in birds from India (99–100% similarity) [34]. The lineage detected in the present study is new and may be a novel lineage from red junglefowl. We revealed that the known and novel lineages found in this study have biological transmission in China and can be transmitted to other birds.

## Conclusion

Using a PCR-based molecular approach, the present study revealed the high prevalence (74.8%) and species of avian haemosporidians in red junglefowl of different sex and age from Yunnan Province, southwestern China. Three species (*H. enucleator*, *L. californicus*, and *P. juxtanucleare*) were identified. This is the first record of avian haemosporidian infection in red junglefowl in China, which extends the host range and genetic diversity of avian haemosporidians and has implications for the control of avian haemosporidia infection in red junglefowl.

## Data Availability

The datasets supporting the findings of this article are included within the article. Representative nucleotide sequences obtained in this study were deposited in the GenBank under accession numbers OM965002–OM965004.

## Abbreviations

*cytb*: Cytochrome *b* gene
ORs: Odds ratios
CIs: Confidence intervals
PCR: Polymerase chain reaction
